# Electrospun Nanofibrous Mesh Based on PVA, Chitosan, and Usnic Acid for Applications in Wound Healing

**DOI:** 10.3390/ijms241311037

**Published:** 2023-07-03

**Authors:** Alexandra Elena Stoica (Oprea), Delia Albuleț, Alexandra Cătălina Bîrcă, Florin Iordache, Anton Ficai, Alexandru Mihai Grumezescu, Bogdan Ștefan Vasile, Ecaterina Andronescu, Florica Marinescu, Alina Maria Holban

**Affiliations:** 1 Department of Science and Engineering of Oxide Materials and Nanomaterials, Faculty of Applied Chemistry and Materials Science, University Politehnica of Bucharest, 011061 Bucharest, Romaniadelia_albulet@yahoo.com (D.A.); ada_birca@yahoo.com (A.C.B.); anton_ficai81@yahoo.com (A.F.); grumezescu@yahoo.com (A.M.G.); 2 Department of Preclinical Sciences, Faculty of Veterinary Medicine, University of Agronomic Sciences and Veterinary Medicine of Bucharest, 105 Blvd. Splaiul Independentei, 050097 Bucharest, Romania; floriniordache84@yahoo.com; 3 National Research Center for Micro and Nanomaterials, University Politehnica of Bucharest, 060042 Bucharest, Romania; bogdan.vasile@upb.ro (B.Ș.V.); alina.m.holban@bio.unibuc.ro (A.M.H.); 4 Research Institute of the University of Bucharest—ICUB, University of Bucharest, 050657 Bucharest, Romania; florica.marinescu@bio.unibuc.ro; 5 Academy of Romanian Scientists, Ilfov Str. No. 3, 50044 Bucharest, Romania; 6 Department of Microbiology and Immunology, Faculty of Biology, University of Bucharest, 91-95 Splaiul Independentei Street, 077206 Bucharest, Romania; 7 Research Center for Advanced Materials, Products and Processes, University of Bucharest, 060042 Bucharest, Romania

**Keywords:** electrospinning, nanofibers, wound healing, PVA, chitosan, usnic acid

## Abstract

Injuries and diseases of the skin require accurate treatment using nontoxic and noninvasive biomaterials, which aim to mimic the natural structures of the body. There is a strong need to develop biodevices capable of accommodating nutrients and bioactive molecules and generating the process of vascularization. Electrospinning is a robust technique, as it can form fibrous structures for tissue engineering and wound dressings. The best way of forming such meshes for wound healing is to choose two polymers that complement each other regarding their properties. On the one hand, PVA is a water-soluble synthetic polymer widely used for the preparation of hydrogels in the field of biomedicine owing to its biocompatibility, water solubility, nontoxicity, and considerable mechanical properties. PVA is easy to subject to electrospinning and can offer strong mechanical stability of the mesh, but it is necessary to improve its biological properties. On the other hand, CS has good biological properties, including biodegradability, nontoxicity, biocompatibility, and antimicrobial properties. Still, it is harder to electrospin and does not possess as good mechanical properties as PVA. As these structures also allow the incorporation of bioactive agents due to their high surface-area-to-volume ratio, the interesting point was to incorporate usnic acid into the structure as it is a natural and suitable alternative agent for burn wounds treatment which avoids an improper or overuse of antibiotics and other invasive biomolecules. Thus, we report the fabrication of an electrospun nanofibrous mesh based on PVA, chitosan, and usnic acid with applications in wound healing. The obtained nanofibers mesh was physicochemically characterized by Fourier transform infrared spectroscopy (FT-IR) and scanning electron microscopy (SEM). In vitro biological assays were performed to evaluate the antimicrobial properties of the samples using the MIC (minimum inhibitory concentration) assay and evaluating the influence of fabricated meshes on the *Staphylococcus aureus* biofilm development, as well as their biocompatibility (demonstrated by fluorescence microscopy results, an XTT assay, and a glutathione (GSH) assay).

## 1. Introduction

Over the past decades, nanotechnology has been providing novel technologies which enable the manufacturing of nanomaterials with unique properties for various applications [1,2,3,4]. One of the most simple, versatile, and effective fabrication methods used in nanotechnology since 1990 is represented by the electro-hydrodynamic (EHD) technique named electrospinning. Since then, substantial progress has been made in the design of the instrument, processing materials, and final products [5,6,7]. Over the past years, the amount of research on electrospun nanofibrous materials for potential ecological, electronic [8,9,10,11], biological, and biomedical applications has increased exponentially [5,6,12,13,14]. This procedure enables the fabrication of nanofibers of sizes ranging from tens of nanometers or microfibers that present diameters of hundreds of micrometers by using a simple principle and an easily controllable apparatus [12,13,15,16,17]. An important advantage of electrospinning is that the morphology, internal structure, sizes, architectures, and surface properties are tunable and controllable, making this technique eligible for various investigations and applications in the biomedical field. Owing to these suitable characteristics, electrospinning has shown a great potential to obtain products with improved and innovative facilities in tissue engineering [18,19,20,21,22], wound dressings [23,24,25,26], drug delivery systems [19,27,28,29,30,31], biosensors [30,32,33], healthcare products [34,35,36], etc.

The most important parameters that influence the fibers’ characteristics are (i) the processing parameters, such as solution feeding rate, needle diameter, rotating speed of the collector, and applied voltage; (ii) the solution properties, such as the polymer’s molecular weight, concentration [15], conductivity, and viscosity; and (iii) the environmental factors such as humidity and temperature. These factors have an important influence on the morphology and structure of fibers [12,37,38].

Due to their characteristics, polymers are great candidates that provide special features to the fabricated nanofibers or scaffolds. About 100 different polymers have already been used for electrospun nanofibers fabrication [12,15,37,39].

Polyvinyl alcohol (PVA) belongs to the category of water-soluble synthetic polymers, being a good candidate for the preparation of hydrogels [40,41,42,43] and widely used in the field of biomedicine [44,45] due to its suitable characteristics such as its biocompatibility, water solubility, nontoxicity, and considerable mechanical properties [41,46,47]. Other uses are related to textiles, papermaking, and coatings [48,49]. Among the properties that make PVA interesting for various applications are easy preparation, good adhesive, and emulsifying properties, a film-forming ability, important mechanical stability, outstanding chemical resistance, and high barrier properties for oxygen and odor [50].

Chitosan (CS) presents impressive biological properties, including biodegradability, nontoxicity, biocompatibility, and antimicrobial properties, promoting pain relief and blood clotting [51], which make it eligible for biomedical applications [52,53,54,55]. The CS structure of nanofibers mimics the natural ECM of the skin and boosts the healing process [46,56]. Electrospinning of CS presents some limitations due to its polycationic nature in solution, as forming solid hydrogen bonds constrains the free motility of the polymeric chain. In contrast, repulsive forces among ionic groups avoid creating enough chain entanglements [46,57,58,59,60].

Usnic acid (UA) has been widely researched in the field of healthcare, being a useful agent in burns and wound therapies [61,62]. Studies showed that UA has an antimicrobial effect against anaerobic bacteria such as *Bacteroides ruminicola* ssp. *Brevis*, *Bacteroides fragilis*, *Bacteroides vulgatus* [63,64], *Clostridium perfringens* [63,64,65], *Propionibacterium acnes* [63,64,65], *Bacteroides thetaiotaomicron* [63,64], Gram-positive bacteria such as *Enterococcus faecium* [63,64], *Enterococcus faecalis* [63,64,65], *Staphylococcus aureus* [64,65,66,67], *S. mutans* [68,69,70,71], *S. epidermidis* [63,65,72], *Streptococcus pyogenes* [63,64,73,74], *Streptococcus pneumoniae* [64,65], Gram-negative bacteria such as *Escherichia coli*, *Haemophilus influenza*, *Pseudomonas aeruginosa*, and Mycobacteria such as *M. aurum*, *M. avium*, *M. smegmatis*, *M. tuberculosis* var. *bovis*, *M. tuberculosis* var. *homini* [64]. Although all these properties make it suitable as a wound-healing agent, there are some limitations in terms of its low water solubility that researchers have tried to overcome by using more soluble salt forms, encapsulating them into liposomes in collagen and gelatin films, including them into β-cyclodextrins, or encapsulating them in electrospun fibers [61].

The purpose of this study was to design and characterize an electrospun nanofibrous mesh based on polyvinyl alcohol, chitosan, and usnic acid with applications in wound healing. The obtained nanofibers are physicochemically characterized using Fourier transform infrared spectroscopy (FT-IR) and scanning electron microscopy (SEM). In vitro biological assays are performed in order to evaluate the antimicrobial properties of the samples, as well as their biocompatibility using fluorescence microscopy, an XTT assay, and a glutathione (GSH) assay.

## 2. Results and Discussions

In the past 20 years, electrospinning has grown significantly as a micro- and nanofiber processing method. It continues to represent the most effective method to produce them in a variety of designs for tissue engineering and regenerative medicine, providing modern and efficient solutions for current day-to-day challenges [75,76,77,78].

### 2.1. Fourier Transform Infrared Spectroscopy (FT-IR)

**Figure 1:**
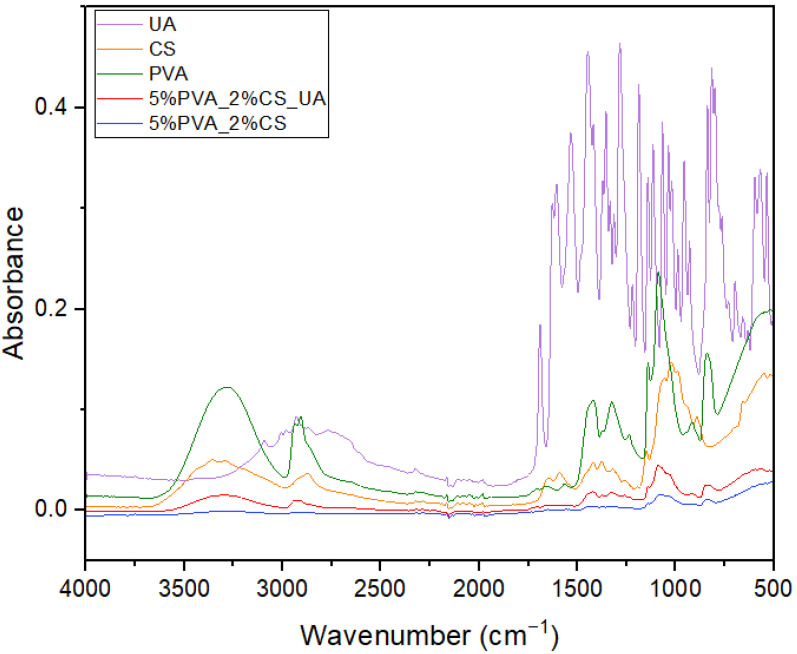
FT-IR spectra of PVA, CS, UA, and 5%PVA_2%CS and 5%PVA_2%CS_UA electrospun nanofiber meshes.

To investigate the composition of the obtained electrospun materials (by identifying the characteristic absorption bands and the interactions between compounds), FT-IR spectra were recorded for the 5%PVA_2%CS and 5%PVA_2%CS_UA nanofibers. In Figure 1, the spectra of raw materials and prepared samples are presented. The absorption band at 3271.67 cm^−1^ corresponds to the O-H stretching and the intramolecular hydrogen bonds of PVA [79,80,81]. An absorption band can be observed at around 2907.87 cm^−1^ and can be attributed to C-H symmetric and asymmetric stretch vibrations for both PVA and CS [79,81]. The peak available at 1645 cm^−1^ can be assigned to the C=O stretching of amide I, and the peak at 1325 cm^−1^ can be assigned to the C-N stretching of amide III, both characteristic of the residual N-acetyl groups from chitosan. Moreover, 1589 cm^−1^ corresponds to the N-H bending of the primary amine [82]. The peak depicted at 1418.97 cm^−1^ can be assigned to the CH_3_ symmetrical deformation vibration of PVA.

The presence of the H-C-O groups of PVA was confirmed by the bands at around 1073.01 cm^−1^, which could also be assigned to the C-O stretching vibration of CS. Characteristic peaks at around 834.82 cm^−1^ are related to the saccharide structure of CS. In the band interval ranging from 515.97 cm^−1^ to 469.60 cm^−1^, the C-C skeleton vibration of PVA can be found [80,83,84,85]. No relevant IR bands characteristics of UA were identified in the fibrous mats due to the fact that the amount of UA present in the samples was under the detection limit of the ATR equipment. The UA presence in the sample was further confirmed by the antimicrobial evaluation.

### 2.2. Scanning Electron Microscopy (SEM)

**Figure 2:**
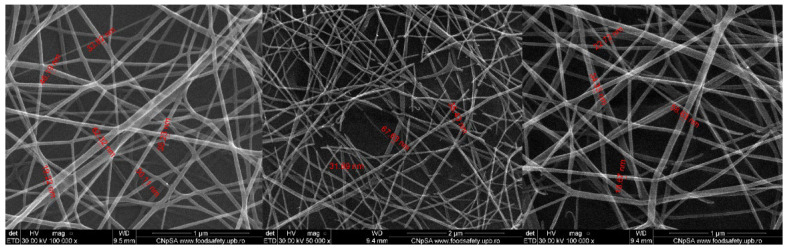

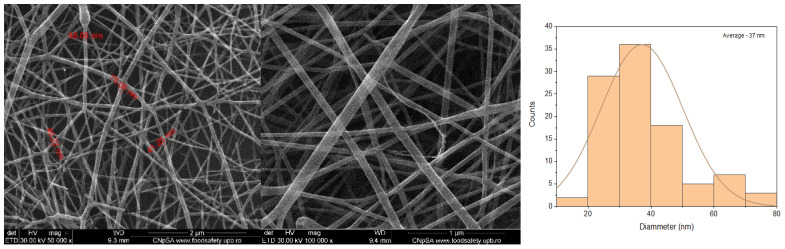
SEM images of electrospun nanofibers obtained for the 5%PVA_2%CS nanofiber mesh.

SEM micrographs showed relevant information regarding the morphology and topography of both simple (5%PVA_2%CS_UA) and usnic acid-incorporated nanospun (5%PVA_2%CS_UA) microfiber meshes. Figure 2 displays the micrographs of the mesh containing PVA and CS. The nanofibrous architecture can be clearly observed, and the formation of an entangled structure of nanofibers was confirmed. Nanofibers with diameters ranging from 14.86 nm to 75.06 nm with predominant sizes between 30 and 40 nm were formed in a nonwoven assembly, having a linear shape and being organized with a random orientation. The images exposed fibers with ramifications that formed a highly porous structure suitable for their application in wound healing, as it resembled the natural ECM.

**Figure 3:**
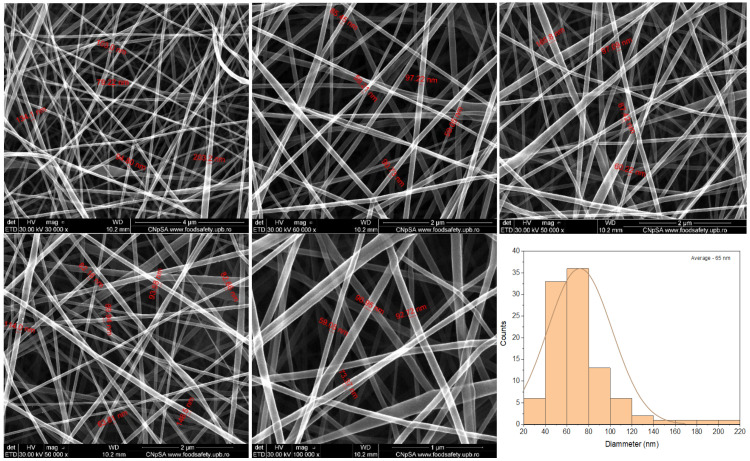
SEM images of electrospun nanofibers obtained for the 5%PVA_2%CS_UA nanofiber mesh.

Figure 3 presents the morphology of the PVA and CS mesh with usnic acid (5%PVA_2%CS_UA), confirming the successful construction of a nanofibrous structure. The nanofibers’ diameter size was between 28 nm and 203.2 nm, with average values at 60–80 nm. It is visible that the nanofiber size increased by introducing usnic acid into the system. However, all other properties, such as the high porosity degree and good structural integrity, remained the same for the simple mesh. These nanofibers were also arranged in a nonwoven assembly. Fibers were smooth and continuous, being arranged in a disordered manner.

### 2.3. Stability Tests

**Figure 4:**
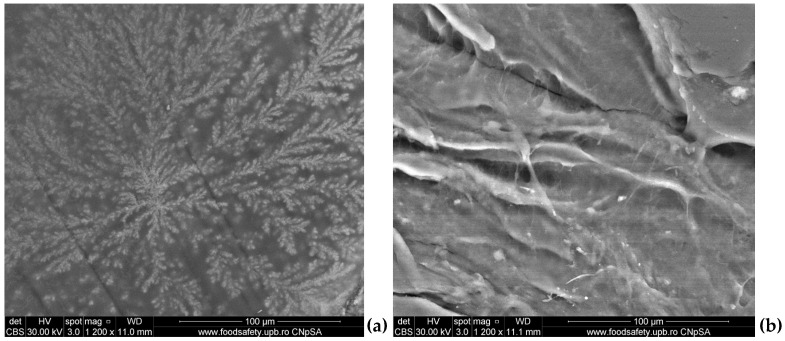
SEM images of fibrous mats (without UA) after immersion in: (**a**) SBF for 72 h, (**b**) PBS for 72 h.

To mimic the stability of the 5%PVA_2%CS and 5%PVA_2%CS_UA fibrous mats in physiological fluids, the samples were immersed in a simulated fluid of blood plasma obtained according to Kokubo’s method [86], termed simulated blood fluid (SBF), and also in phosphate buffer (PBS). As can be observed in Figure 4, the SEM images revealed a good interaction of SBF and PBS with the fibrous mats, highlighting that on the surface of the samples, mineral deposits appeared. Moreover, no fibrillar structures were available due to the ability of the tested mats to swell in various aqueous environments.

### 2.4. XTT Assay

**Figure 5:**
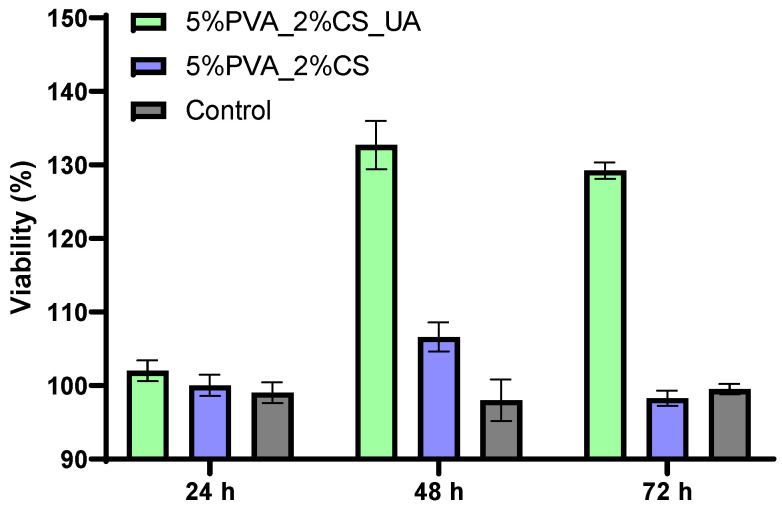
XTT assay after exposure of cells to the 5%PVA_2%CS_UA and 5%PVA_2%CS nanofibers mesh.

The XTT test graph shown in Figure 5 delivers information about the cell viability after exposure to the 5%PVA_2%CS and 5%PVA_2%CS_UA samples at 24 h, 48 h, and 72 h. Both samples presented good biocompatibility; the cell viability degree was above 90% at all three measurement times. After the first 24 h, there were no significant changes, showing that the two materials had similar values to the control. After 48 h, there was an increase in cell viability for the 5%PVA_2%CS sample, which exceeded the control values. However, after 72 h, a considerable decrease could be registered, which implied that the material without usnic acid did not have a proliferative effect on cells.

Nevertheless, the PVA/CS mesh with usnic acid incorporated (5%PVA_2%CS_UA), showed extraordinary results after 48 h and 72 h, increasing the cell viability by almost 30% compared to that of the control. This fact confirmed the suitable biocompatibility of the electrospun mesh with usnic acid and made the material eligible as a proper biodevice that sustains the proliferation and growth of cells. Moreover, these findings support previous studies that indicated the protective role of usnic acid in endothelial cells by exhibiting analgesic, antipyretic, and anti-inflammatory characteristics [87,88].

### 2.5. Glutathione (GSH) Assay

**Figure 6:**
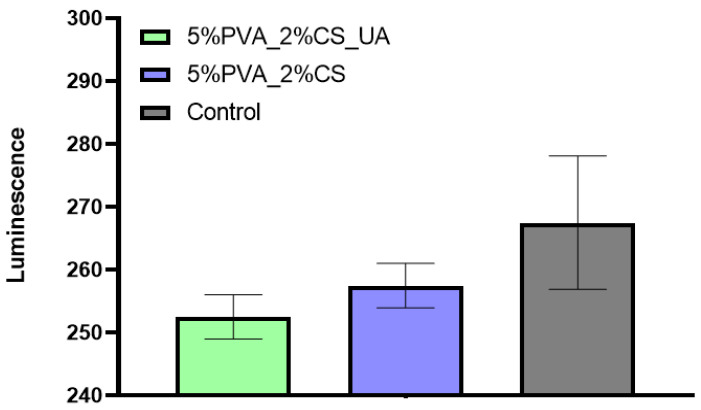
GSH Assay for AFSC after being in contact with the 5%PVA_2%CS and 5%PVA_2%CS_UA meshes.

The oxidative stress of AFSC was evaluated by the amount of glutathione existing for the 5%PVA_2%CS and 5%PVA_2%CS_UA nanofiber meshes versus the control, as shown in Figure 6. Both 5%PVA_2%CS and 5%PVA_2%CS_UA meshes exhibited a glutathione amount comparable with that of the control, the differences being statistically insignificant. Therefore, results indicated that in contact with the AFSC, the materials did not stimulate the occurrence of oxidative stress. High levels of GSH are correlated with cellular damage and are very important in order to assess the level of cellular stress that can potentially lead to apoptosis and cell death.

### 2.6. Fluorescence Microscopy

**Figure 7:**
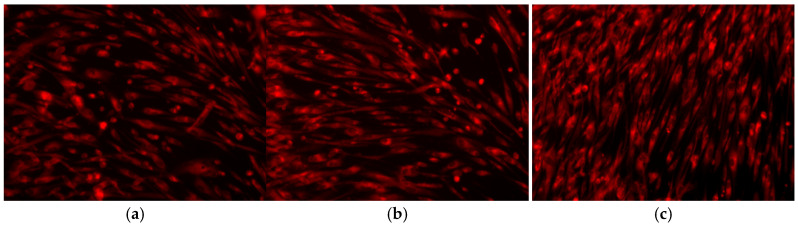
Fluorescence micrographs for (**a**) 5%PVA_2%CS mesh; (**b**) 5%PVA_2%CS_UA mesh, and (**c**) control.

AFSC cultivated in the presence of 5%PVA_2%CS and 5%PVA_2%CS_UA were observed by fluorescence microscopy, as shown in Figure 7. The high number of viable (red) cells demonstrated the biocompatibility of the fabricated fibrous mats [87]. Cellular morphology was compared with control cells. The AFSC cultivated with nanofibrous mashes presented a normal fibroblast-like phenotype. The AFSC presented a good attachment to the substrate compared with the control, with the integrity of the cellular membrane and a low number of vacuoles, suggesting that the nanofibrous meshes did not exhibit a cytotoxic effect. Additionally, studies [89] have shown that the process triggered by usnic acid does not include DNA damage, indicating that usnic acid is not a genotoxic compound.

### 2.7. Effect of Nanostructured Meshes on Biofilm Production

**Figure 8:**
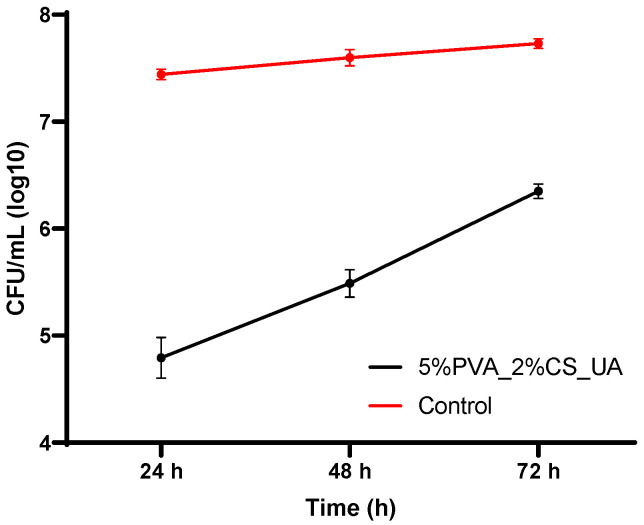
The mean value of log10 CFU (colony-forming units)/mL of *S. aureus* recorded at 24 h, 48 h, 72 h for the 5%PVA_2%CS_AU mesh compared to the control.

Many serious and resistant infections in hospitalized patients are caused by the opportunistic pathogen *S. aureus*. The growth curve graph shown in Figure 8 presents the CFU/mL versus incubation time at 24 h, 48 h, and 72 h for *S. aureus* treated with the 5%PVA_2%CS_AU mesh. Data represent the mean values of two determinations performed at each time point. It is visible that the 5%PVA_2%CS_AU mesh managed to have an improved anti-biofilm activity, showing lower rates of CFU/mL than the control. However, it is noticeable that in time, this activity decreases.

However, as Figure 6 reveals, the anti-biofilm effect was maintained for at least 72 h against *S. aureus*, cultured in standard conditions. The biofilm inhibition could be evaluated by the release of active UA contained in the prepared nanofiber meshes, a natural compound that has been previously reported to act as an antimicrobial agent against Gram-positive species such as *S. aureus* [87,90,91,92,93,94] and *E. faecalis* [92] and Gram-negative species such as *P. aeruginosa* and *E.coli* [92]. The ability of usnic acid to interfere with the growth and biofilm formation of *S. aureus* constitutes an important aspect in developing alternative antistaphylococcal therapies containing natural compounds. Compared to conventional antibiotics, these medicines have the significant advantage of being less toxic and preventing the spread of bacteria that are resistant to treatment [87].

Over the past decades, many developments have focused on fabricating ECM-like scaffolds made of nanofibers through electrospinning because of their unique morphology, high surface-area-to-volume ratio, and porosity [95,96]. ES-fabricated biostructures provide good assistance for various tissue types regarding regeneration and reconstruction, namely, they are able to mimic the natural ECM and initiate special biological responses, accelerating the attachment and stimulating cell growth, proliferation, and differentiation [24,97].

Given the very good reaction of PVA in contact with the biological environment and its ability to significantly absorb high quantities of water or biological fluids without dissolving, it means that the best solution is to blend PVA with other polymers to obtain a favorable wound dressing that highlights the advantages of both polymers [50]. At the same time, CS is more suitable to be electrospun with other polymers in order to efficiently develop the properties of the fibers [98,99,100,101,102]. The selection of a flexible polymer in combination with CS, such as PVA, can be chosen in order to improve the electrospinning of chitosan and the properties of the end product [57,103,104,105]. One important factor in the design of CS-based biomaterials is represented by the mechanisms of its degradation in the biological environment [106]. Numerous synthesis methods have been studied to create CS, which degrades over days, weeks, or even months, depending on the needs of the specific application or biodevice [105,106,107,108]. Furthermore, studies [109,110,111,112] reported that by adding bioactive components to functionalized nanomaterials, accelerated healing was achieved by regulating the metabolic pathway and growth factors. Usnic acid (UA), a dibenzofuran produced from lichen, is one of them [113]. Usnic acid is able to reduce the inflammation related to burns and to encourage wound closure through keratinocyte monolayers [64,114].

Herein, composite PVA/CS and PVA/CS/UA nanofibrous scaffolds were developed by electrospinning, and their antimicrobial properties, as well as their biocompatibility, were demonstrated using fluorescence microscopy, an XTT assay, and a glutathione (GSH) assay.

On the one hand, the large surface area and pore volume ratio and uniform and well-structured porosity of the fiber mat allowed the wound exudates absorption while reducing the potential bacterial infection [115]. As a general remark, the SEM images recorded for both samples exposed macroporous fibrous networks with ramifications that formed a highly porous structure, uniformly distributed and randomly oriented defect-free fibers with diameters ranging from 14.86 nm to 203.3 nm, suitable for their application in wound healing, as it resembled the natural ECM [116,117]. To mimic the stability of fibrous mats in physiological fluids, the samples were immersed in simulated body fluid (SBF) and also in phosphate buffer (PBS), respectively. The SEM images revealed a good interaction of SBF and PBS with fibrous mats, underlining that on the surface of samples, mineral deposits became visible. Furthermore, no fibrillar structures could be observed due to the ability of the tested mats to swell in different aqueous environments. It is important to mention that the swelling ability of the PVA/CS fibers represents an important property that characterizes the utilization of the mats for wound dressing applications [118].

On the other hand, given that cell viability was above 90% at each of the three measurement times, both samples exhibited good biocompatibility. There were no noticeable changes after the first 24 h, indicating that the two materials exhibited values that were comparable to those of the control. Furthermore, it was noticed that in contact with the AFSC, the fibrous materials did not promote the occurrence of oxidative stress. High levels of GSH are linked to cellular damage and are highly significant in order to evaluate the level of cellular stress that can potentially lead to apoptosis and cell death. Moreover, the high number of viable cells demonstrated the biocompatibility of the fabricated fibrous structures (Figure 7).

Last but not least, because an important part of serious and resistant infections in hospitalized patients is induced by the opportunistic pathogen *S. Aureus* [119], the 5%PVA_2%CS_AU mesh was evaluated in order to assess the effect of the nanostructured meshes on biofilm production. The results proved that the mesh presented an improved anti-biofilm activity, demonstrating lower rates of log CFU/mL than those of the control; however, it was clear that, in time, this activity decreased.

Thus, the developed fibrous mats based on PVA/CS/UA hold great promise in wound-healing applications.

## 3. Materials and Methods

### 3.1. Materials

Polyvinyl alcohol (PVA, (C_2_H_4_O)_n_, 99% hydrolyzed), chitosan (CS, medium molecular weight, 75–85% deacetylation degree), and usnic acid (98% purity) were purchased from Sigma-Aldrich (Merck Group, Darmstadt, Germany). All the reagents were analytical grade and used without any further purification.

### 3.2. Preparation Methods and Electrospinning Parameters

In the first stage, two solutions of 5%PVA and 2%CS were prepared. Thus, a first solution of 5%PVA (Mw) in water was prepared. The solution was maintained at 80 °C under gentle stirring for 1 h, followed by ultrasonication. Then, a second 2%CS solution was made using aqueous acetic acid 1N at room temperature under gentle stirring for 1 h to form a homogeneous solution, which was subjected to ultrasonication as well.

**Figure 9:**
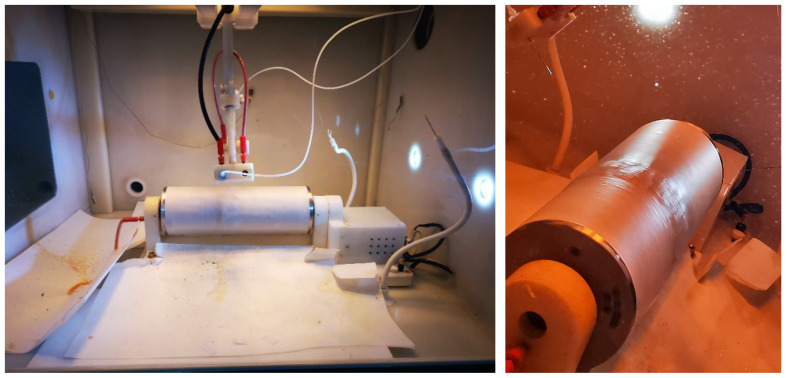
Electrospinning process. The solutions are expulsed in jets through the syringe and form nanofibers on the rotating collector.

The electrospinning process (Figure 9) was carried out using a Tong Li Tech nanofiber electrospinning unit at room temperature. The device is composed of a syringe pump, a high-voltage DC power supply, a spinning polymer solution, and a rotating cylindrical collector.

Thus, for the sample containing PVA and CS (named 5%PVA_2%CS), 5 mL of solution 1 (5%PVA) and 5 mL of solution 2 (2%CS) were mixed and loaded into a polypropylene syringe connected to an 18-gauge blunt-end needle and then mounted on a digital syringe pump. The electrospinning procedure was carried out using the specific operation parameters shown in Table 1. The needle-to-target distance was 120 mm. The fibers were directly deposited on a piece of grounded aluminum foil.

In order to obtain a nanofiber mesh based on PVA, chitosan and usnic acid (named 5%PVA_2%CS_UA), solution 1 (5%PVA) and solution 2 (2%CS) were prepared through the same method as previously described. Additionally, a third solution of 1% usnic acid in dimethyl sulfoxide (DMSO) was prepared. Further, 4.5 mL of solution 1 (5%PVA) together with 4.5 mL of solution 2 (2%CS) and 1 mL of solution 3 (1% usnic acid) were mixed and loaded into a polypropylene syringe connected to an 18-gauge blunt-end needle and then mounted on a digital syringe pump. The electrospinning deposition was carried out under the operating conditions described in Table 1. The needle-to-target distance was 120 mm. The fibers were directly deposited on a piece of grounded aluminum foil.

### 3.3. Physicochemical Characterization

#### 3.3.1. Fourier Transform Infrared Spectroscopy (FT-IR)

Infrared spectroscopy is a useful technique that can offer valuable information about interactions in polymer blends, confirming the integrity of functional groups characteristic of the synthesized materials. FT-IR spectra were recorded using the Nicolet 6700 FT-IR spectrometer, purchased from Thermo Nicolet (Madison, WI, USA). A small amount of particulate suspension was analyzed by a ZnSe crystal, and measurements were carried out through 32 sample scans between 4000 and 500 cm^−1^ at a resolution of 4 cm^−1^ at room temperature. In order to be able to register the acquired information, the spectrometer was connected to a data acquisition and processing unit through the Omnic program (version 8.2 Thermo Nicolet).

#### 3.3.2. Scanning Electron Microscopy (SEM)

The morphological analyses of the two materials were carried out using scanning electron microscopy. For this purpose, a scanning electron microscope purchased from the FEI Company (Hillsboro, OR, USA) was used. The samples were shaped with a diamond disc and fixed on a sample support to be introduced into the analysis zone. The shape and size of the PVA/CS nanofibers of the mesh were determined through micrographs of the samples by direct measurements of the resulting secondary electron beam with a 30 keV energy at different points of the samples.

#### 3.3.3. Stability Tests

In order to assess the interactions among the obtained fibrous mats (cut in a square shape of 1/1 cm), the samples were immersed in simulated blood fluid (SBF) obtained according to Kokubo’s method [86] and in phosphate buffer (PBS) as well. For 72 h, the fibrous materials were subjected to physiological conditions at 37 °C. After that, qualitative analysis by SEM was performed.

### 3.4. In Vitro Biocompatibility

#### 3.4.1. XTT Assay

The biocompatibility of the synthesized nanofibrous mesh samples was evaluated using an XTT reagent (2,3-Bis-(2-Methoxy-4-Nitro-5-Sulfophenyl) -2H-Tetrazolium- 5-Carboxanilide) according with the manufacturer protocol (CyQUANT™ XTT Cell Viability Assay Kit, Thermo Fischer Scientific, Waltham, MA, USA). The assay kit included the XTT reagent and an electron-coupling reagent. The XTT reagent is a tetrazolium-based compound sensitive to cellular redox potential. Actively viable cells convert the water-soluble XTT compound to an orange-colored formazan product. The sensitivity and consistency of the assay are significantly increased when used with the electron-coupling reagent. The human mesenchymal amniotic fluid stem cells (AFSC) were grown in DMEM (Sigma-Aldrich, Saint Louis, MO, USA) supplemented with 10% fetal bovine serum, 1% antibiotics (penicillin and streptomycin) (Sigma-Aldrich, Saint Louis, MO, USA), changed twice a week. The AFSC were placed in 96-well plates, at a density of 3000 cells/well, in the presence of nanofibrous mesh for 24 h, 48 h, and 72 h. The control samples were represented only by AFSC cultivated in the same condition but without the nanofibrous mesh. Subsequently, 70 µL of XTT solution was added to the cells, followed by incubation at 37 °C for 4 h. After a vigorous homogenization of the formazan crystals, the absorbance was read at 450 nm using a TECAN Infinite M200 spectrophotometer (Männedorf, Switzerland).

#### 3.4.2. GSH-Glo Glutathione Assay

The oxidative stress was assessed using the GSH-Glo glutathione assay kit (Promega, Madison, WI, USA). AFSC was seeded at a density of 3000 cells in 300 µL DMEM supplemented with 10% fetal bovine serum and 1% antibiotics (penicillin, streptomycin/neomycin) in 96-well plates. After 24 h of seeding, AFSC was put in contact with the nanofibrous mesh and then incubated for another 24 h. Furthermore, 100 µL of 1X GSH-Glo reagent was added, followed by an incubation at 37 °C for 30 min, followed by 100 µL of luciferin detection reagent and an incubation at 37 °C for an additional 15 min. The medium from the cell cultures was well homogenized, and then the plate was read on the luminometer (Microplate Luminometer Centro LB 960, Berthold, Germany).

#### 3.4.3. Fluorescence Microscopy

For the evaluation of cellular biocompatibility in terms of cellular shape, attachment to the substrate, the integrity of the cellular membrane, and the number of vacuoles, we used fluorescence microscopy and a red CMTPX fluorophore (Thermo Fischer Scientific, Waltham, MA, USA). The CMTPX is a cell tracker for the long-term tracing of living cells, which, when entered into the cells, start to emit fluorescence. The viability and morphology of the AFSC were evaluated after 5 days of cultivation of the AFSC in the presence of the nanofibrous mesh. The CMTPX fluorophore was added to the cell cultures at a concentration of 5 µM and incubated for 30 min. Finally, the AFSC cultures were washed with PBS and visualized by fluorescent microscopy using an Olympus CKX 41 digital camera driven by CellSense Entry software (Olympus, Tokyo, Japan).

#### 3.4.4. Anti-Biofilm Effect

To test the effect of the nanostructured meshes on the biofilm production, samples were sterilized by exposure to UV radiation for 20 min on each side. A 0.5 cm/0.5 cm sample of the material was individually deposited in a well of a 6-well sterile plate. Over the deposited materials, 2 mL of liquid medium was added to the wells, followed by 50 μL of 0.5 McFarland microbial suspension. The 6-well plates thus prepared were incubated at 37 °C for 24 h. After incubation, the materials were washed with PBS (phosphate-buffered saline), and the medium was changed to allow biofilm development. Plates were incubated for different periods (24 h, 48 h, 72 h). Upon expiration of the incubation period, the sample on which the biofilm was developed was washed with PBS and placed in a sterile tube containing 1 mL of PBS. The tube was vortexed vigorously for 30 s to detach the cells from the developed biofilm. The obtained cell suspension was diluted, and various dilutions were seeded on solidified culture media plates to obtain and quantitate the number of colony-forming units per mL.

## 4. Conclusions

Electrospinning offers a robust and versatile technique to produce 2D and 3D nanofiber meshes with patterned and porous structures. Numerous opportunities in the field of biomedicine have opened up thanks to the biomimicry that these structures offer, including in scaffolds, drug delivery, cell behavior control, and regenerative medicine. Incorporating bioactive molecules into electrospun nanofiber meshes represents a beneficial development in wound healing because these hybrid structures positively affect wounds and can initiate and accelerate the healing process. This study presented the preparation and characterization of an electrospun nanofiber mesh based on polyvinyl alcohol, chitosan, and usnic acid with applications in wound healing. The physicochemical characterization revealed a fibrous structure with fiber dimensions ranging from 14.86 nm to 75.06 nm with predominant sizes between 30 and 40 nm. SEM images showed a nonwoven assembly having a linear shape with a random orientation. The images exposed fibers with ramifications that formed a highly porous structure suitable for their application in wound healing because it mimicked the natural ECM. The XTT assay showed extraordinary results after 48 h and 72 h for 5%PVA_2%CS_UA, increasing the cell viability by almost 30% compared to that of the control. This fact confirmed the suitable biocompatibility of the electrospun mesh with usnic acid and made the material eligible as a proper biodevice that sustains the proliferation and growth of cells. The cell viability revealed using fluorescent microscopy was comparable with that of the control, meaning that the nanofiber meshes did not exhibit a cytotoxic effect. From the point of view of biofilm development, the 5%PVA_2%CS_UA presented an improved anti-biofilm activity against the *S. aureus* strain. In conclusion, all these results affirm that electrospun nanofibers mesh based on PVA, chitosan, and usnic acid could be a promising material for applications in wound healing.

## Figures and Tables

**Table 1:** Electrospinning Operating Parameters.

Parameters	Solutions	
	5%PVA_2%CS	5%PVA_2%CS_UA
Flow rate, mL/h	3–7	5
Output voltage, KV	−6 -> −3 17 -> 20	−6.5 17
Fan speed, rpm	80	80
Humidity degree, %	~20	~20
Temperature, °C	~28	~28
Heater output, KW	0.6	0.6

## Data Availability

Not applicable.
